# The chemical armament of reef-building corals: inter- and intra-specific variation and the identification of an unusual actinoporin in *Stylophora pistilata*

**DOI:** 10.1038/s41598-017-18355-1

**Published:** 2018-01-10

**Authors:** Hanit Ben-Ari, Moran Paz, Daniel Sher

**Affiliations:** 1 Department of Marine Biology, Leon H. Charney School of Marine Sciences, University of Haifa, Haifa, Israel; 2 The Interuniversity Institute for Marine Sciences, Eilat, Israel

## Abstract

Corals, like other cnidarians, are venomous animals that rely on stinging cells (nematocytes) and their toxins to catch prey and defend themselves against predators. However, little is known about the chemical arsenal employed by stony corals, despite their ecological importance. Here, we show large differences in the density of nematocysts and whole-body hemolytic activity between different species of reef-building corals. In the branched coral *Stylophora pistillata*, the tips of the branches exhibited a greater hemolytic activity than the bases. Hemolytic activity and nematocyst density were significantly lower in *Stylophora* that were maintained for close to a year in captivity compared to corals collected from the wild. A cysteine-containing actinoporin was identified in *Stylophora* following partial purification and tandem mass spectrometry. This toxin, named Δ-Pocilopotoxin-Spi1 (Δ-PCTX-Spi1) is the first hemolytic toxin to be partially isolated and characterized in true reef-building corals. Loss of hemolytic activity during chromatography suggests that this actinoporin is only one of potentially several hemolytic molecules. These results suggest that the capacity to employ offensive and defensive chemicals by corals is a dynamic trait within and between coral species, and provide a first step towards identifying the molecular components of the coral chemical armament.

## Introduction

Coral reefs are the largest biogenic structures on Earth: they reach sizes of >1000 km, form extreme “hotspots” of biodiversity, and provide numerous ecosystem, community and economic resources. As their name attests, the physical structure of most coral reefs is composed of hermatypic (reef-building) stony corals. As a result of anthropogenic stress and global climate change, hermatypic corals are increasingly facing challenges such as loss of zooxanthellae due to bleaching^1,2^, increased microbial loads^3–8^, competition with macroalgae^9^, and the potential for nutrient limitation and loss of exoskeleton due to ocean acidification^10^. Under each of these scenarios, reef-building corals may need to rely even more on offensive or defensive chemicals to survive in a changing world^2,11^.

Stony corals belong to the phylum cnidaria, which also includes sea anemones, jellyfish, and hydrozoans. Cnidarians are soft-bodied, morphologically simple organisms, and many of them (e.g. corals) are either sessile or have only limited mobility. Nevertheless, most cnidarians are predators^2,12^, and they use a combination of a sophisticated delivery system (the stinging cells, nematocytes^13–15^) and complex and potent venom to catch prey such as zooplankton, larger crustaceans and even fish^16,17^. In addition, cnidarians use allomonal chemistry to protect themselves against predators, microbial pathogens and fouling organisms^5,6,18,19^, as well as to compete for space with other reef organisms^9,20,21^. Finally, toxin-like compounds may be involved in endogenous physiological roles such as neural and endocrine signaling, development and prey digestion (e.g.^22,23^). Thus, cnidarians depend to a large extent on their impressive chemical arsenal for survival.

The toxic and bioactive arsenal of cnidarians has been intensively studied for over 30 years^24^, with most studies focused on two main groups of cnidarians and their associated chemical armament: the venom of sea anemones and the defensive compounds of soft corals and gorgonians^25^. Anthozoan venom is comprised mostly of proteins and peptides, many of which are short neurotoxins affecting ion conductance^26–28^, hemolysins^29^ and phospholipase A2s^30,31^. Most of the compounds derived from soft corals can be defined as “secondary metabolites” (rather than proteins or peptides), and are often sterols and terpenes^32,33^. Octocorals are a rich source of antimicrobial, antifouling and cytotoxic compounds likely involved in defense, allelopathy or predator deterrence^32,33^. Importantly, studies on the diverse chemical arsenal of cnidarians have resulted in novel compounds for medical, agricultural or biotechnological uses^34–36^.

Perhaps surprisingly, very little is known about the chemical arsenal employed by stony corals for survival despite their sheer numerical abundance, biomass, and ecological importance as keystone organisms in coral reefs. The lack of studies of coral toxins may be due to the apparent lack of toxicity of most corals or to the difficulty in obtaining sufficient biomass for biochemical work from these often-protected organisms. With the exception of a phospholipase A2 toxin from the “fire coral” *Millepora* ^37,38^ (which is a hydrozoan, and thus not a true scleractinian coral) and small cysteine-rich peptide toxins from *Acropora millepora* recombinantly expressed in bacteria^39^ to date no toxins have been isolated or characterized from reef building corals. Nevertheless, early studies described widespread, albeit highly variable, toxicity in corals^40,41^, and these organisms catch prey^2,12,42^, defend themselves from predators^43^, resist microbial infections^5,6,44^ and are involved in fierce chemically-mediated competition for space^20,45,46^.

To better understand venom diversity and activity in reef-building corals, we compared two manifestations of this armament, namely the abundance of nematocysts and the hemolytic activity contained in the coral tissue, among abundant reef-building corals in the Gulf of Aqaba. After identifying consistent differences between hermatypic corals, we focused further studies on *Stylophora pistillata*, one of the most abundant corals in the Indo-Pacific and a commonly used model for studying various aspects of coral physiology, genetics and ecology (e.g.^47–53^). In particular, we assessed to what extent the number of nematocysts and hemolytic activity differ within colonies of this branching coral. Finally, using bioassay-guided column chromatography and mass spectrometry, we identify a new group of coral-derived hemolysins belonging to the actinoporin family.

## Results

### Coral species differ widely in nematocyst density and tissue hemolytic activity

**Figure 1:**
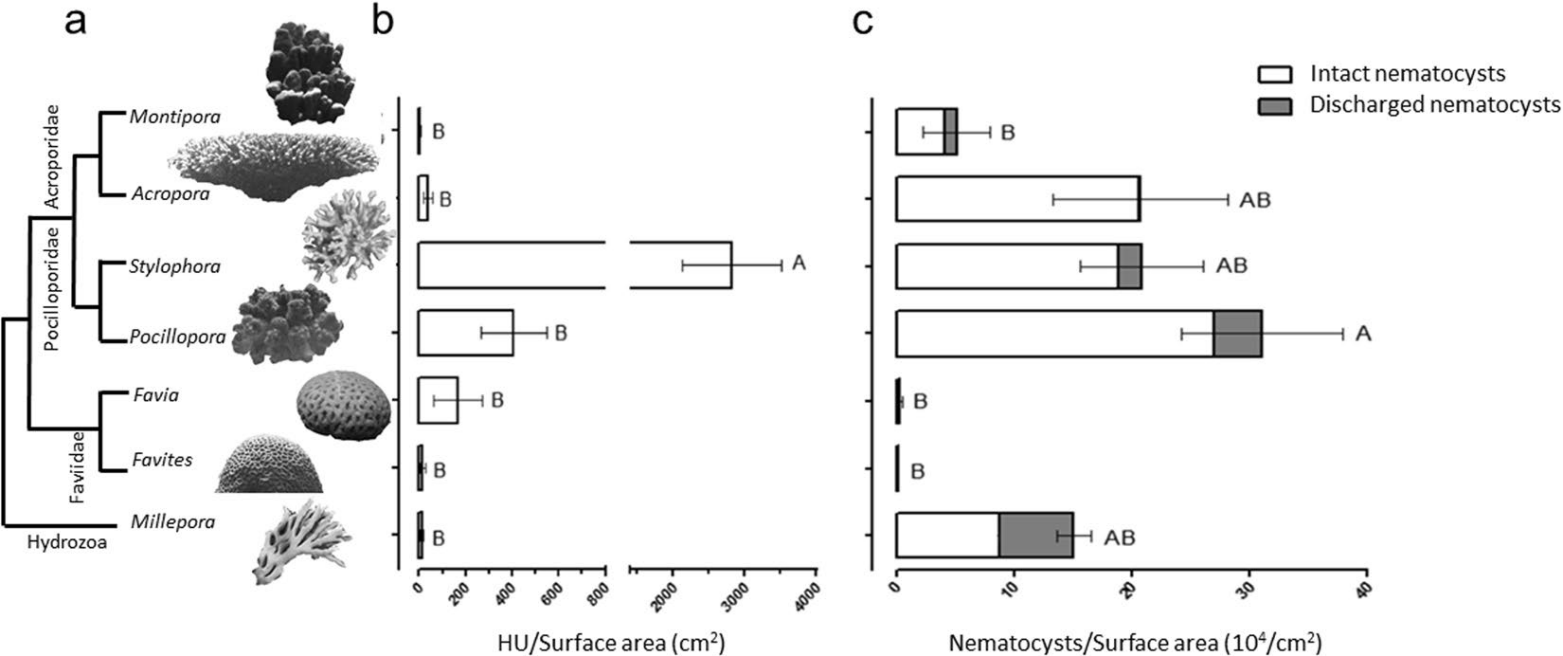
Inter-species variability in hemolytic activity and nematocyst density. (**a**) Schematic phylogeny of the seven studied reef-building corals^77,78^. The images are not shown to scale. (**b**) Hemolytic activity in tissue extracts of the seven corals, normalized to surface area. HU-Hemolytic Unit, defined as the amount of hemolytic activity causing 50% hemolysis of human red blood cells in a standard, 500 μl reaction (see materials and methods). (**c**) Nematocyst density in the tissue, normalized to surface area. Data in panels C and D are the mean ± SE of one fragment (branch tip) each from 5 different colonies.

To assess the extent to which reef-building corals produce and deliver toxins, we collected fragments from seven abundant reef-building organisms (six anthozoan corals and the hydrozoan “fire coral” *Millepora sp*) from the Gulf of Aquaba (N 29° 30.211′ E 34° 55.068), at the northern tip of the Red Sea. For clarity, we refer to all these organisms, including *Millepora* sp, as “corals”, using the genus name throughput the manuscript. As shown in Fig. 1, large differences were seen in the hemolytic activity and the nematocyst numbers (normalized to coral surface area) between the different corals, and these differences may be related to their phylogeny (Fig. 1a). The highest hemolytic activity was found in *Stylophora* (2818.55 + 693.95 HU/cm^2^), seven-fold higher than the organism with the second highest activity, the closely related *Pocillopora sp*. (407.07 + 141.38 HU/cm^2^, Fig. 1b). The highly-hemolytic *Stylophora* contained more than 600-fold higher hemolytic activity than the coral with the lowest activity, *Montipora* (4.64 + 2.55 HU/cm^2^). Examination of total nematocyst density in the sample tissue revealed a somewhat different pattern. The branching corals (Pocilloporidaee and Acroporidae) had far more nematocysts per surface area than the robust corals (the latter belonging to the family Favidae), with the tissue of *Pocillopora* containing ~350-fold more nematocysts than that of *Favites* (30.966 + 6.83 10^4^/cm^2^ vs 0.093 + 0.03 10^4^/cm^2^, respectively). Despite using an intense tissue extraction protocol (air-brushing the coral followed by homogenization), most of the nematocysts visible by microscope were not discharged (Fig. 1c). This raises the question of whether the hemolytic activity of the tissue homogenate originates from the nematocysts (e.g.^54^). Given that the highest levels of hemolysis were found in *Stylophora*, we focused on this coral for subsequent analyses.

### Intra-colonial variation in hemolytic toxicity of *Stylophora*

**Figure 2:**
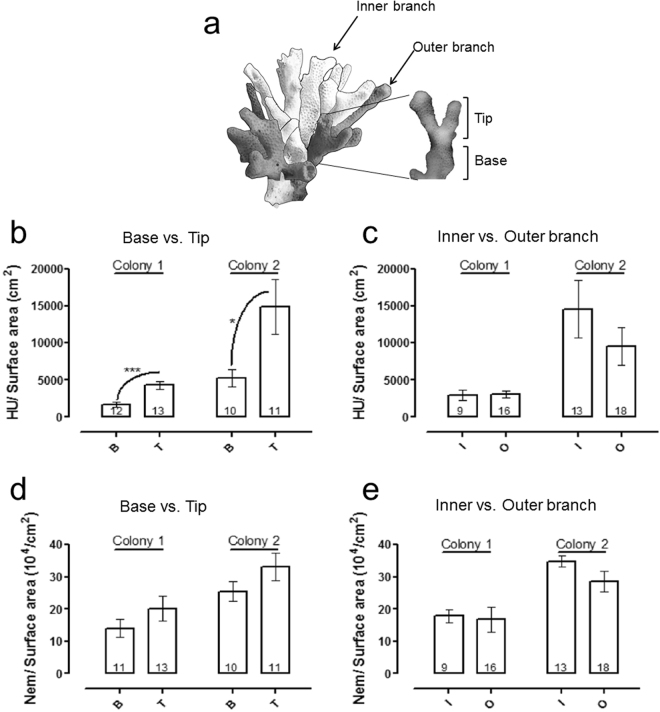
Intra-colonial differences in toxicity in *Stylophora*. (**a**) A skeleton of *Stylophora*, schematically illustrating the location of the inner (white) and outer (grey) branches, as well as the difference between branch base and tip. (**b**,**c**) Comparison of the hemolytic activity, normalized to surface area, between fragments from different locations in the two *Stylophora* colonies. (**d**,**e**) same as (**b**), but for number of nematocysts. Numbers below the bars represent number of replicates, error bars indicate mean ± SE. Asterisks indicate significant differences (p = 0.0003 for colony 1 and p = 0.0293 for colony two, using Tukey-Kramer HSD test).

Coral colonies are composed of individual polyps, each of which may be exposed to different conditions such as light intensity, water flow and accessibility to prey or predators (e.g.^55,56^). It is thus conceivable that the chemical armament of the coral will also be differentially distributed within the colony. To test this hypothesis, two *Stylophora* colonies (colony 1 and 2) were divided into fragments differing in their physical location within the colony (Fig. 2a), and the nematocyst density and hemolytic activity were measured for each fragment. In general, colony 2 demonstrated higher hemolytic activity and slightly higher nematocyst density than colony 1 (Fig. 2b and c). Within each colony, the same pattern was observed: similar levels of hemolytic activity and nematocyst densities were found between outer and inner branches but the branch tips exhibited significantly higher hemolytic activity than the branch bases (p = 0.0003 for colony 1 and p = 0.0293 for colony two, One-Way ANOVA).

**Figure 3:**
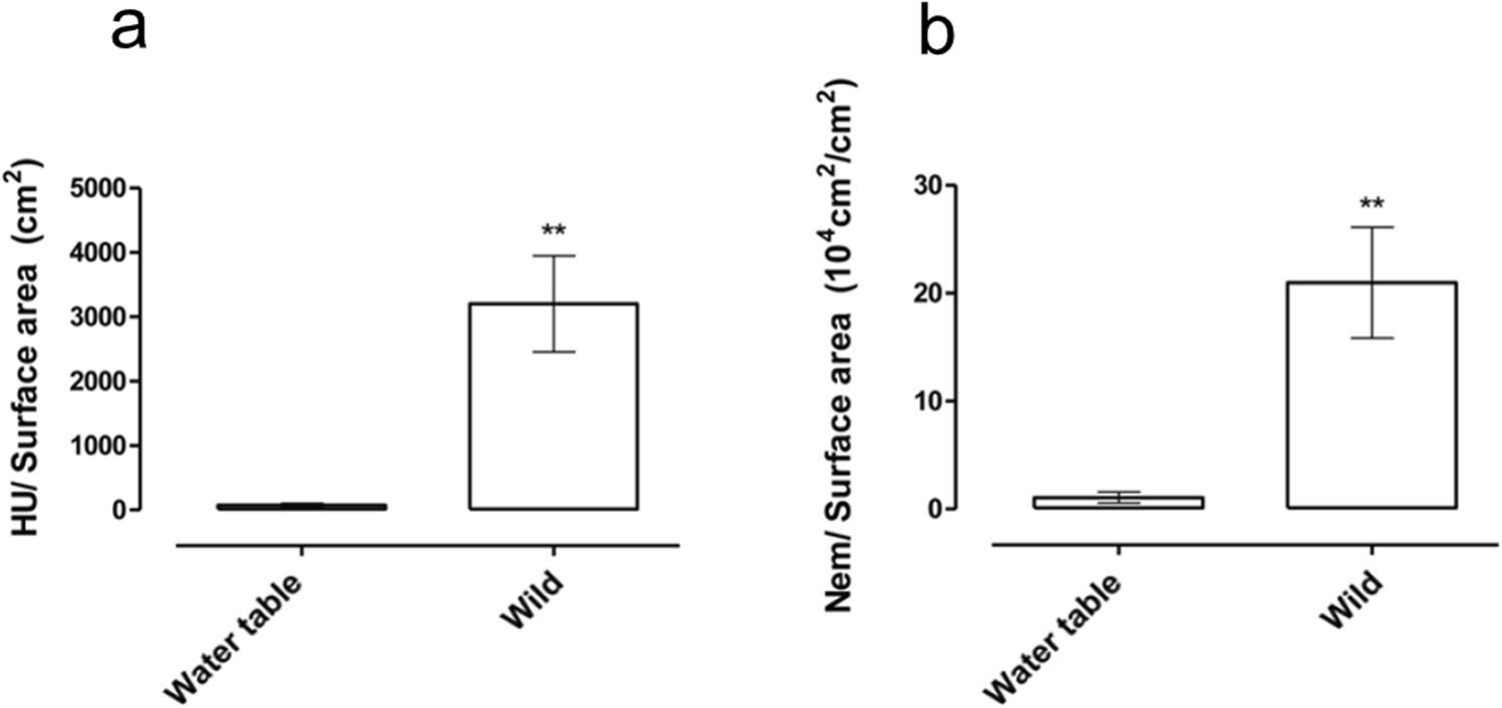
Lower hemolytic activity in *Stylophora* fragments acclimated to laboratory water tables. A comparison is shown between the numbers of hemolytic units (HU) found in the tissue (**a**) and the number of nematocysts (**b**), both normalized to the sample surface area of wild and acclimated fragments. N = 5. Error bars indicate standard error about the mean with 95% confidence limits attached. Asterisks indicate that significantly difference (P < 0.05, using Tukey-Kramer HSD test).

Many studies aiming to understand the response of corals to changing conditions include a preliminary stage where corals are collected from the field, acclimated to laboratory conditions, and only then subjected to experimental manipulations (e.g.^51,57^). Aiming to perform controlled experiments to assess how the chemical armament of *Stylophora* changes in response to environmental conditions, we collected several colonies from the wild, divided them into fragments and maintained the fragments for close to a year in open water tables to acclimate them to the experimental conditions. However, the hemolytic activity and nematocyst densities in the fragments maintained in the water tables were significantly lower than those of fragments collected from wild animals (20–100 fold, Fig. 3, the “wild” fragments are those shown in Fig. 1). The fragments maintained in the water tables looked healthy to the naked eye (e.g. extended polyps could be observed), and were able to catch and feed on *Artemia salina* nauplii^44^.

### An actinoporin hemolysin from *Stylophora*

We next sought to isolate and characterize the hemolytic substance(s) in the *Stylophora* extract using bioassay-guided fractionation. Initial tests, aimed to select the appropriate method for fractionation using column chromatography, revealed that in all tested methods (gel filtration, anion exchange and hydrophobic interaction chromatography) the hemolytic activity partitioned into at least two distinct peaks, differing by their molecular mass, charge or hydrophobicity (not shown). While each of the chromatography methods was able to separate at least part of the hemolytic activity from the majority of the protein content of the extract, most of the hemolytic activity (>80% in all cases) was lost upon chromatography, regardless of the method used. This was not due to changes in salinity, as the hemolytic activity of the crude extract was not strongly affected by changes in salinity (0.2–1.4 M NaCl). The loss of activity during chromatography (>80%) was higher than that occurring when the crude extract was incubated for 6-8 hours at 4 °C (~50%), conditions similar to those used for the chromatography.

**Figure 4:**
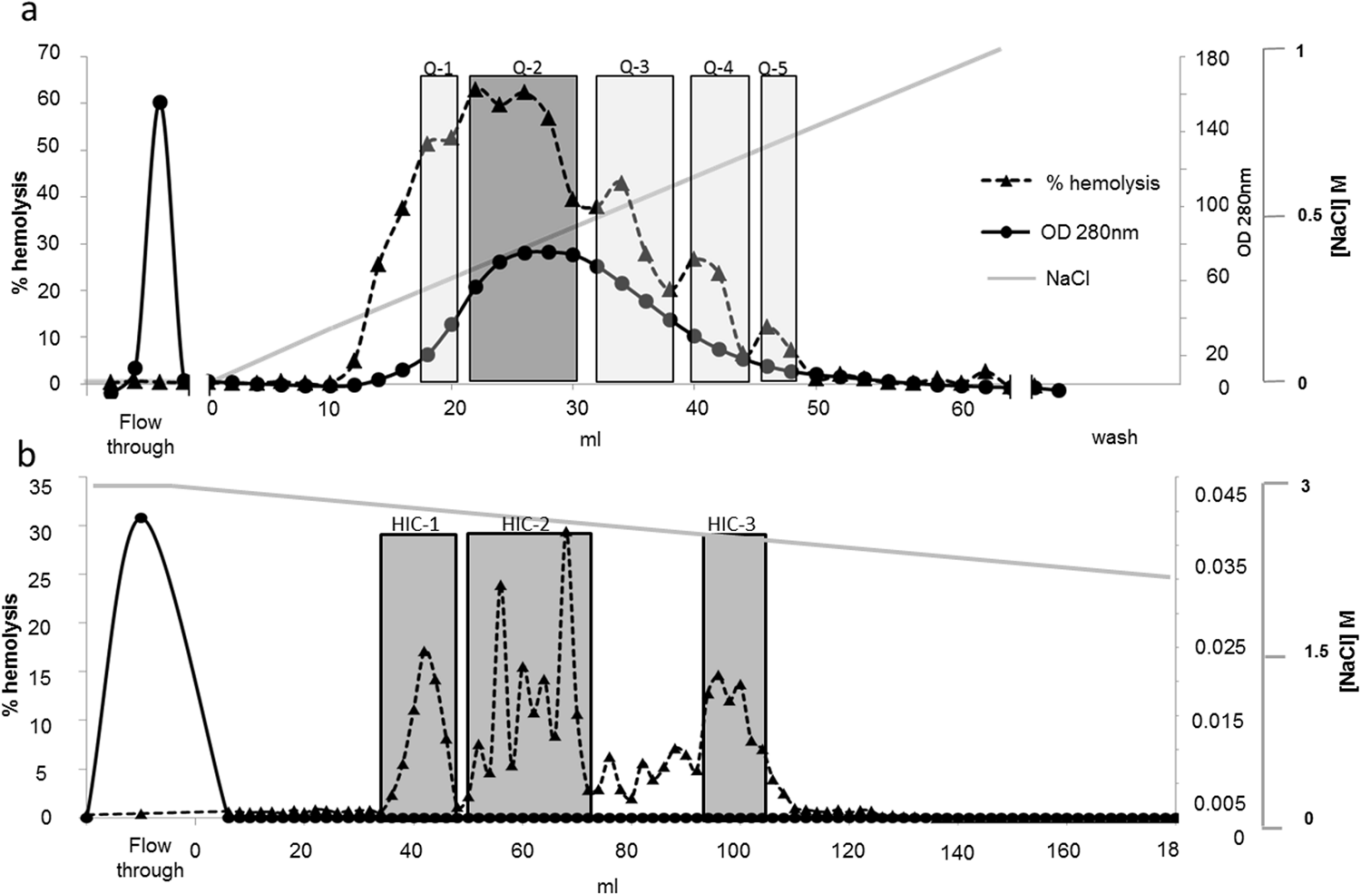
Separation and partial purification of an actinoporin-like hemolysin from *Stylophora*. In the chromatograms, the shaded areas represent active fractions that were pooled and submitted either to re-chromatography (after anion exchange) or two tandem mass spectrometry (after hydrophobic interactions chromatography). The straight grey lines represent the gradient of salt used for chromatography. (**a**) Separation of *Stylophora* crude extract by anion exchange chromatography on a Q- sepharose FF 18.5 ml column. (**b**) Separation of pool Q-2 from panel A, above, on a Hydrophobic Interactions Phenyl- Sepharose HP 7.8 ml column.

Based on these preliminary tests, we decided to use two stages of chromatography, anion exchange followed by hydrophobic interactions, to isolate and identify the major hemolytic compound(s) in the *Stylophora* crude extract. As shown in Fig. 4a, a broad and irregular peak in hemolytic activity was observed following separation by anion exchange, again suggesting the presence of several hemolytic compounds. The combined hemolytic yield of the major hemolytic fractions, Q-1 to Q-5, represented 22% of the total hemolytic activity in the crude extract loaded on the column. The fractions corresponding to hemolytic peak Q-2 were pooled and subjected to hydrophobic interactions chromatography (HIC), resulting in at least three distinct hemolytic peaks (Fig. 4b). The combined hemolytic yield of peaks HIC-1 to HIC-3 was <1% of what was loaded on the column. The fractions comprising each of these three hemolytic peaks were pooled and analyzed by tandem mass spectrometry (MSMS), resulting in the identification of 5-12 proteins in each pool (Table 1). Thus, at this stage, the putative hemolysin was not completely purified. Nevertheless, the most abundant protein in each of these pools (comprising 48-90% of the total ion current) was identified as an actinoporin with high identity (60%) to the hemolytic toxin Sticholysin-2 from the sea anemone *Stichodactyla helianthus* ^58^ (see below). There was no other protein shared among all three pools. These results strongly suggest that the actinoporin is the hemolysin responsible for the majority of the hemolytic activity in these pools. We thus propose the rational nomenclature of Δ-Pocilopotoxin-Spi1 (Δ-PCTX-Spi1) for this hemolysin, the first hemolytic toxin to be partially isolated and characterized in true reef-building corals^59^.

**Table 1:** Proteins identified in the three hemolytic fractions after HIC using shotgun tandem mass spectrometry, ordered by the peak area in each fraction (a semi-quantitative estimate of protein abundance). The actinoporin is shown in bold.

Protein name	Pool number	Peak Area	Function	Identity	Query Cover	E- value	Organism
**m.2523**	**1**	**1.764E8**	**Sticholysin 2**	**60%**	**88%**	**2E-70**	***Stichodactyla helianthus***
m.1230	1	3.952E7	Hypothetical Protein (Myosin 10 like)	27%	50%	2E-17	*Nematostella vectensis*
m.7445	1	2.023E7	uracil-DNA glycosylase	38%	78%	2E-40	
m.2226	1	1.998E7	golgi-associated plant pathogenesis-related protein 1-like	42%	70%	7E-17	*Cynoglossus semilaevis*
m.12035	1	1.046E7	hypothetical protein (Polysaccharide deacetylase-like protein)	43%	74%	8E-102	*Nematostella vectensis*
m.24455	1	8.602E6	gelsolin	63%	97%	9E-46	*Suberites domuncula*
m.6423	1	7.527E6	Hypothetical protein (serine hydroxymethyltransferase, mitochondrial)	77%	88%	0.0	*Xenopus (Silurana) tropicalis*
m.2128	1	4.522E6	protein disulfide-isomerase A6	63%	96%	0.0	*Strongylocentrotus purpuratus*
m.1877	1	4.345E6	iron/zinc purple acid phosphatase-like protein	62%	97%	2E-157	*Chrysemys picta bellii*
m.1271	1	4.388E6	caspase 3-like protein	99%	100%	0.0	*Stylophora pistillata*
m.20013	1		transmembrane protease serine 6-like	42%	47%	3E-64	*Thamnophis sirtalis*
m.21014	1		beta-catenin	71%	98%	0.0	*Chaetopterus variopedatus*
**m.2523**	**2**	**3.490E8**	**Sticholysin 2**	**60%**	**88%**	**2E-70**	***Stichodactyla helianthus***
m.2226	2	2.132E7	golgi-associated plant pathogenesis-related protein 1-like	42%	70%	7E-17	*Cynoglossus semilaevis*
m.1027	2	9.580E6	annexin A6-like	36%	100%	2E-129	*Biomphalaria glabrata*
m.1230	2	6.079E6	Hypothetical Protein (Myosin 10 like)	27%	50%	2E-17	*Nematostella vectensis*
m.6423	2	2.112E6	Hypothetical protein (serine hydroxymethyltransferase, mitochondrial)	77%	88%	0.0	*Xenopus (Silurana) tropicalis*
m.7409	2	5.158E5	predicted protein (glutamate dehydrogenase)	71%	98%	0.0	*Sunxiuqinia dokdonensis*
m.11815	2		hypothetical protein (peroxiredoxin-1)	86%	71%	2E-36	*Branchiostoma floridae*
**m.2523**	**3**	**7.349E7**	**Sticholysin 2**	**60%**	**88%**	**2E-70**	***Stichodactyla helianthus***
m.3179	3	5.966E7	nuclear receptor coactivator 5-like	39%	50%	1E-53	*Saccoglossus kowalevskii*
m.1594	3	1.277E7	predicted protein (glutamate dehydrogenase)	71%	99%	0.0	*Nematostella vectensis*
m.11815	3	3.320E6	hypothetical protein (peroxiredoxin-1)	86%	71%	2E-36	*Branchiostoma floridae*
m.24936	3	3.307E6	predicted protein (Glucosamine-6-phosphate isomerase 1)	74%	90%	6E-158	*Nematostella vectensis*
m.4166	3	4.704E5	predicted protein (catenin alpha-2-like isoform X4)	61%	735	0.0	*Nematostella vectensis*

**Figure 5:**
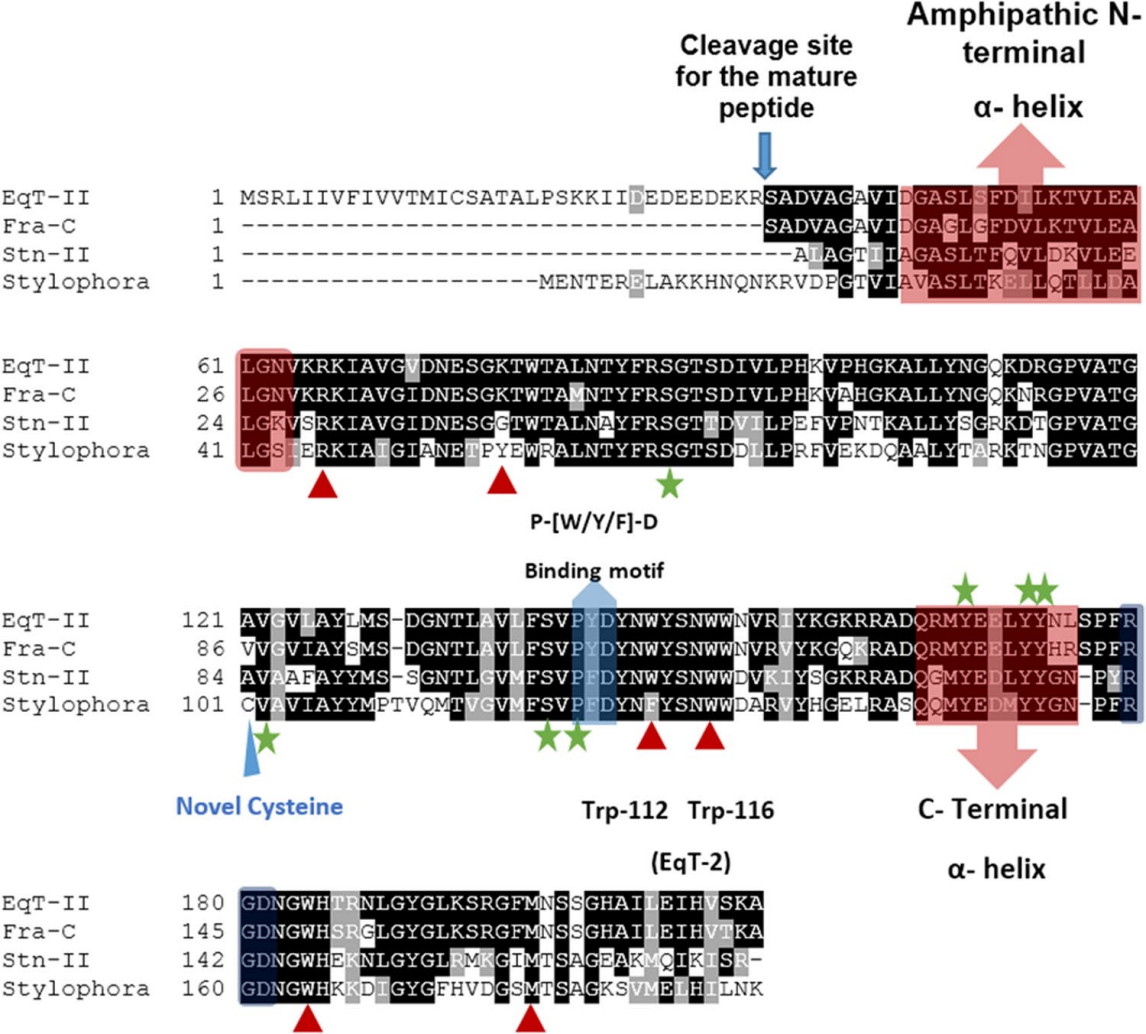
Multiple alignment of the *Stylophora* actinoporin and three well studied sea anemone actinoporins: equinatoxin II (EqT-II, *Actinia equina*), stycholysin II (Stn-II, *Stichodactyla helianthus*) and fragaceatoxin C (Fra-C, *Actinia fragacea*). The regions highlighted in red are the C and N- terminal α- helixes, the red triangles point out residues that have shown to be important for membrane attachment^61,69^, the blue arrow shows the RGD motif involved in oligomerization^105^ and the green stars represent residues that have shown to be important for sphingomyelin recognition^58,61^. The blue region shows the conservation of the P-[W/Y/F]-D membrane binding motif^106,107^.

Actinoporins have been intensively studied to understand their hemolytic mode of action and as models for protein-membrane interactions. The structures of three actinoporins have been elucidated, and the roles of many amino acid residues are known (recently reviewed by^60–62^). As shown in Fig. 5, the *Stylophora* actinoporin, Δ-Pocilopotoxin-Spi1, shares many functional features with the model actinoporins equinatoxin II (Eqt-II)^63,64^, sticholysin II (Stn-II)^58^ and Fra-C^65^ (from the sea anemones *Actinia equina* and *Stichodactyla helianthus*, and *Actinia fragacea* respectively). First, the coral actinoporin is predicted to have two α-helices, one at the N-terminus (involved in pore formation) and one at the C-terminus, similar to sea anemone actinoporins^61^. Many of the residues previously shown to be involved in membrane recognition and hemolysis are conserved in Δ-Pocilopotoxin-Spi1 (Fig. 5 ^58,66–69^). Interestingly, Trp112 in Eqt-II, which was shown to be critical for sphingonmyelin recognition and hemolytic activity^69^, is replaced in Δ-Pocilopotoxin-Spi1 by phenylalanine, which is also hydrophobic. This important residue is replaced in other actinoporins, for example by leucine in HALT-1 from *Hydra magnipappilata* ^70–72^. A notable difference between the anemone and coral actinoporins is that Δ-Pocilopotoxin-Spi1 has a single Cysteine residue (marked with a blue triangle in Fig. 5). Most actinoporins lack cysteines with the exception of HALT Toxins and some actinoporins from *Heteractis magnifica* ^71,73^. These results support the identification of Δ-Pocilopotoxin-Spi1 as the protein responsible for the hemolysis in the active fractions after chromatography.

### Actinoporins are common in corals

**Figure 6:**
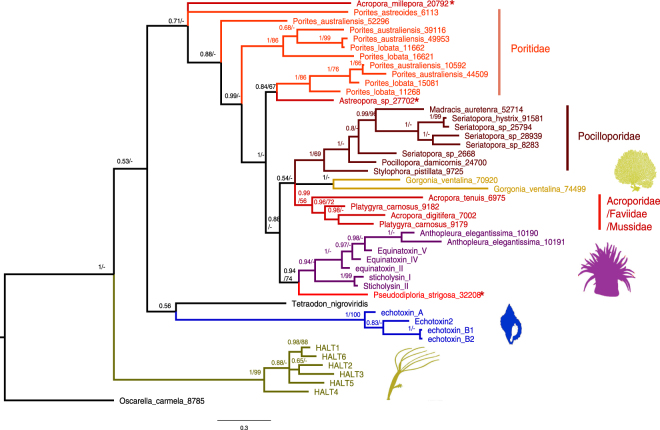
Phylogeny of actinoporins and actinoporin-like proteins in cnidarians. A Bayesian tree is shown, with numbers on the nodes representing posterior probabilities and Maximum Likelihood bootstrap values (when above 50%). Schematic images represent (from top to bottom) gorgonians, sea anemones, molluscs and hydra. All of the sequences not originating from these animals are from scleractinian corals, and are colored based on the clades/families mentioned in the text (*represents sequences from Acroporidae and Mussidae that do not cluster with the other sequences from this group, as described in the text). The sequences for the proteins in this tree can be found in the Supplementary fasta file.

Actinoporins are common in sea anemones and Hydra, yet are missing from the transcriptome of at least one scyphozoan jellyfish^16,72^. Similar proteins are found also in other non-cnidarian organisms, with the role of most of these non-cnidarian actinoporins currently not known^72,74,75^. Recently, a database containing the genomes and/or transcriptomes of 20 coral species has been assembled^76^, allowing the presence of genes encoding this family of hemolysins to be mapped across coral diversity. We could identify genes or transcripts encoding actinoporins in all but one of the coral datasets (*Acropora hyacinthus* was the exception), with 1-6 different sequences identified per transcriptome (Supplementary Table 1). As shown in Fig. 6, the phylogeny of actinoporins was generally congruent with that of the organisms themselves, with Hydra, sea anemones and corals each found mostly as a different monophyletic group on the phylogenetic tree. This suggests that a single actinoporin-like gene was present in ancestral cnidarians (before the split between Hydrozoans and Anthozoans) that was may have been lost in some lineages and duplicated in others. Actinoporins from two coral families, Pocilloporidae and Poritidae, mostly cluster separately, in agreement with phylogenetic analyses using other molecular markers which suggest that these two lineages separated over 200 million years ago (Ma), in the Triassic era^77,78^. In contrast, some actinoporins from the *Platygyra* and *Acropora* clustered together, despite these two lineages belonging to distinct families (Faviidae and Acropopridae) that also separated ~200 Ma. In addition, an actinoporin from *Astreopora sp*. (also an acroporid coral) clearly clustered with those from the Porites lineage, and one from *Pseudodiploria strigosa* clustered with the sea anemone proteins. This suggests that the gene duplication and diversification may have occurred at several different times during cnidarian evolution.

## Discussion

### Differences between corals in aspects of their chemical armament

The use of chemical armament (toxins, either secreted or injected as venom) is considered critical to the survival of the producing organism. Venoms and toxins, as well as their delivery systems, are often considered to be under strong selective pressure (e.g.^39,79,80^), and it is thus reasonable to hypothesize that differences in the potency of the chemical armament may be related to the relative importance of this armament in the biology (life history) of the producing organisms (e.g.^81^). In this study, we provide the first quantitative measurements of two aspects of the chemical armament of reef-building corals, namely the density of the nematocysts (the venom delivery systems) and the hemolytic activity of the coral tissue. Neither of these measures is related exclusively to the chemical armament of the organism: besides venom delivery, nematocysts can also entangle prey^23,82^, possibly assist digestion^83,84^ and, in Hydra, facilitate locomotion^82^. Similarly, while hemolysins are commonly found in venoms, including those of cnidarians, similar proteins may be involved in prey digestion, immunity and development^22,23^. In the following discussion, we assume that the nematocysts and hemolytic activity are traits related to the chemical armament of the corals, noting that additional experiments are necessary to test this assumption.

The six reef-building corals and the hermatypic hydrozoan (*Millepora*) studied here clearly differed in both aspects of their chemical armament, with the Pocilloporid corals (*Stylophora* and *Pocillopora*) having the highest hemolytic activity and among the highest nematocyst counts per unit surface area. Previous studies have documented high variability in the toxicity of different stony corals, however no systematic trends were observed^40,41^. This may be due to the less quantitative nature of the toxicity methods previously employed (e.g. hemolysis was not quantified in terms of hemolytic units per fragment size). The same quantitative approach enabled us to identify intra-colonial differences in hemolytic activity between the tips of the branches and their bases. The branch tips of stony corals have previously been shown to have slightly different transcriptome patterns compared to branch bases, including differences in the expression of genes involved in nematocyst differentiation^56^. It is tempting to speculate that the differences in hemolytic activity between the branch tips and bases are related to their exposure to the external environment, including prey and predators and competitors.

Are the nematocysts and hemolytic toxins used for prey capture, predator deterrence or territorial competition? It is unlikely that either the nematocysts or the hemolytic activity assayed here function in territorial competition, since *Stylophora* and *Pocillopora*, the corals with the highest hemolytic activity and nematocyst density, are considered weaker than *Favia* in the hierarchy of coral aggressiveness when competing for space on the reef^45^. Given the limited number of organisms and physiological conditions assayed here, it is difficult to clearly state whether the hemolysin and nematocysts are correlated with the predatory capacity of the different corals, or to their level of exposure to potential predators. *Stylophora* and *Pocillopora* have relatively high predation rates^85^, and heterotrophy can support a significant part of their metabolism (e.g.^11,12,42,51^), suggesting a role for the nematocysts and hemolytic activity in feeding. However, the predation rate of *Acropora* has been shown to be much lower than that of *Stylophora* and *Pocillopora* ^85^, and while the hemolytic activity of *Acropora* is also much lower, the nematocyst density in these three corals is similar (Fig. 1). Another factor that seems to broadly differentiate between the corals with high and low nematocyst densities is that the polyps of *Stylophora*, *Pocillopora* and *Acropora* in the Gulf of Aqaba are expanded almost all the time, whereas the polyps of *Favia* and *Favites* expand only during the night^86^. Thus, *Favia* and *Favites* may be exposed both to lower predation pressure and to lower prey densities. Finally, the *Stylophora* that were reared in the water tables were not exposed to any predation, and prey densities are also likely lower than on the reef. Taken together, these results support the notion that the hemolytic toxicity and nematocyst density in corals are both dynamic traits related to the defensive and/or offensive (predatory) capability of these organisms.

It is worth noting that, in our study, the Hydrozoan *Millepora sp*. did not emerge as one of the organisms with the highest toxicity, although its nematocyst density was relatively high and a relatively high percent of the nematocysts were discharged during our extraction procedure (Fig. 1). *Millepora sp*. are known to cause painful stings when touched, leading to their common name, “Fire Corals”. Previous studies have shown that fire corals produce phospholipase A2 toxins (PLA2s)^37,38^, and one might expect that these membrane-disrupting enzymes would cause hemolysis. However, while several publications describe the purification or partial purification of PLA2s from cnidarians, none of these proteins have been shown to be hemolytic^87,88^. Our own experiments with commercially-available bee venom PLA2 revealed high phospholipase activity but no hemolysis (not shown). We therefore propose that the *Millepora* PLA2s are not hemolytic, and that, similar to jellyfish and some sea anemones^16^, *Millepora* have lost the ability to produce active hemolysins, with the venom evolving to comprise other toxins with paralytic and/or pain-causing functions.

### Actinoporins and the potential for synergism contributing to the hemolytic activity

Aiming to identify the molecular components responsible for the hemolytic toxicity of the *Stylophora* coral extract, we used bioassay (hemolysis)-guided fractionation of the crude coral extract, observing at least two hemolytic peaks differing in retention times. Since multiple peaks were observed using three different chromatography methods (based on molecular mass, charge and hydrophobicity), these peaks likely represent different hemolysins. An alternative option is that the multiple peaks correspond to different aggregation levels of the actinoporin we eventually identified, or association of the actinoporin with other proteins. We also observed that the majority of the hemolytic activity (>80%) was lost upon column chromatography, regardless of the method used, and that this loss was not due to the time it took to perform the fractionation or to the pH or salinity used. We thus speculate that the hemolytic activity in the coral tissue is due to two or more compounds working in synergism, one of them likely the actinoporin we identified, Δ-Pocilopotoxin-Spi1. Indeed, a transcript encoding part of a second putative actinoporin was identified in the *Stylophora* transcriptome (Supplementary Table 1). Synergistic activity has previously been observed between non-covalently bonded venom constituents in spiders and cone snails^89–91^, involving in some cases also cytolytic peptides^92^. Recently, synergism has been demonstrated between Sticholysins I and II, two actinoporin isoforms isolated from the same sea anemone, *Stichodactyla helianthus*, which form hetero-pores on the target membranes^93^. The initial studies of Equinatoxins, in which these proteins were isolated and identified using bioassay-guided fractionation, do not report on the fraction of the total hemolytic activity represented by the isolated toxins, and thus it is currently unclear whether the same phenomenon, namely loss of activity during purification, also occurs in sea anemones^94,95^. Given the complexity of most animal venoms (which contain tens to thousands of different molecules), the potential for synergistic activity between venom constituents (or constituents of defensive chemical cocktails) is an exciting field of research. This also highlights the importance of quantitative analysis of the activity eluted during chromatography to identify such potential cases of synergism.

### What are the roles of actinoporins in corals

Actinporins have been identified in the nematocysts of sea anemones, Hydra and corals^16,96–98^, and thus some of these proteins at least seem to be ancient bona-fide components of cnidarian venom. In some cnidarians, most notably sea anemones and Hydra, actinoporins are observed as gene families that underwent gene duplication and diversification (Fig. 6 ^60,72^). Unlike many toxins from cone snails and scorpions, which are under strong positive/diversifying selection, most of the residues in actinoporins are under strong negative/purifying selection, although some might be affected by episodic diversifying selection^39,60,80^. Our analysis reveals multiple different actinoporin transcripts, likely resulting from gene diversification events, in some stony corals such as *Porites australiensis*, *Porites lobata*, *Platygyra carnosus* and *Seriatopoa sp*, but not in others (Fig. 6, Supplementary Table 1 and fasta file). Given the fragmented state of most of the cnidarian genomes and transcriptomes, including those we used for this analysis^76^, and the fact that many genome or transcriptome assembly algorithms collapse closely-related gene variants^99^, we cannot rule out similar diversification in the other stony corals shown in Fig. 6. The limited gene duplication and diversification observed in *Stylophora* (only two sequences, one of which is partial, Supplementary Table 1), a trait often suggested to be driven by a “predator-prey arms race” (e.g.^80,100^), together with the relatively low number of nematocysts discharged during the homogenization process (Fig. 1), lead us to speculate that Δ-Pocilopotoxin-Spi1 may be a non-nematocystic protein primarily used for defense against predators.

### Concluding remarks - the flexibility and potential ecological importance of the chemical armament of corals

In this study, we focused on two aspects of the chemical armament of cnidarians, the density of the nematocysts in the tissue (the nematocysts being the delivery system for many toxins) and the tissue hemolytic activity. We propose that these can be considered as quantitative functional traits related to prey capture or defense from predators. Our results reveal large and consistent differences in these two traits, both between stony coral species and within colonies of *Stylophora*. Furthermore, these traits are dynamic over time, as seen in the reduction in both traits in *Stylophora* colonies maintained in water tables compared to colonies from the wild, and we propose that these changes are related to predation pressure. Notably, previous studies have suggested such a possibility; for example, Gochfeld^43^ showed that the Hawaiian coral *Porites compressa* responded to grazing by butterflyfishes by increasing the density of nematocysts, and that these changes were associated with reductions in palatability and subsequent predation rates on the damaged corals.

In some cases, toxins (including hemolysins) can also be secreted^101^. Recently, chemical cues released by healthy corals have been shown to attract the larvae of both fish and corals, whereas degraded corals did not release such cues, or released non-attractive cues^102^, suggesting that such chemical cues are critical for the maintenance of a healthy, reproducing reef. It is not unlikely that the toxins produced by corals may be part of the chemical “bouquet” released by corals and conveying information on the species composition or health of the reef. Further studies of the chemical armament of corals may not only help understand the physiology and ecology of these organisms but may also provide biomarkers of reef health.

## Material and Methods

### Coral collection and maintenance

All coral colonies and coral fragments were collected from an *in situ* coral nursery near the Interuniversity Institute for Marine Sciences (IUI N 29° 30.211′ E 34° 55.068) at a 5–12 m depth located at the Gulf of Eilat, a northeastern extension of the Red Sea (Fig. 1). The use of corals for this research was approved by the Israel Nature and Parks Authority (authorizations 2012/38770, 2014/40464 and 2015/40869).

For the comparison of hemolytic toxicity and nematocyst density between different organisms, five similarly sized coral fragments were collected by SCUBA diving during July 5 2015, maintained during the dive in plastic bags filled with ambient seawater, and immediately transferred to a −80 °C freezer (within several minutes) until use. Each fragment was collected from a different colony. No release of hemolytic activity into the water was observed in preliminary tests where *Stylophora* corals were fragmented under controlled conditions, suggesting that any changes in hemolytic activity are not due to stress during sampling. Fragments from the following organisms were collected: *Stylophora pistillata* (mean surface area of each collected fragment (SA) of 23 ± 4 cm^2^), *Montipora meandrina* (SA of 27 ± 6 cm^2^), *Acropora sp*. (SA of ∼18 ± 5 cm^2^), *Favia favus* (SA of ∼4 ± 6 cm^2^), *Favites sp*. (SA of ∼75 cm^2^), *Pocillopora sp*. (SA of 15 ± 9 cm^2^) and *Millepora sp*. (SA 21 ± 7 cm^2^).

In order to test whether different parts of the colony exhibit similar levels of hemolytic activity, two *Stylophora* colonies were randomly selected and collected on July 2, 2015. The dimensions for Colony 1 were approximately 16 × 14 × 7 cm and for colony 2 were 11 × 9 × 7 cm (length-width-height). The colonies were gently transferred to the IUI wet lab (within several minutes) and cut into fragments using a custom-made small electric saw. Each fragment was given an ID number to identify the fragments’ mother colony and its’ location within the colony (see below) and immediately transferred to a 80 °C freezer until use.

In order to test whether acclimation in water table facility change the hemolytic activity or nematocyst counts, five similarly sized coral fragments of *Stylophora* were collected in September 15, 2014 as described above, and kept for approximately 11 months in an outdoor water table with running natural seawater. The corals experienced natural dark/light regimes and were shaded with a 30% black mesh. The corals were suspended in the water using fine nylon threads to avoid contact with the container walls. On August 9, 2015 the tissue was removed, the hemolytic toxicity and nematocyst density were measured as described below and the results compared to those of the fragments collected during July 2015.

### Tissue extraction, surface area measurement and nematocyst counts

Frozen coral fragments were removed from the −80 °C freezer, the tissue was immediately removed from the skeleton using an air brush and filtered sea water, and the volume of the tissue-water slurry was recorded. The tissue-water slurry was further homogenized using an electrical homogenizer (mrc,HOG-160-1/2) for 10 seconds. 100 μl of the homogenate were then used for nematocysts counting using a hemocytometer (five arbitrarily selected fields per fragment) and the rest was centrifuged at 12,000 rpm for 3 min. The supernatant of this aqueous crude extract was used for the hemolysis assay described below. The remaining skeleton was dried and its surface areas was measured using the nextEngine’s 3D Scanner and ScanStudio™ software (NextEngine, Inc. Santa Monica, California).

### Hemolysis assay

The hemolytic activity of the tissue homogenates was determined as described previously^103^ using human blood type B+ blood obtained from the Eilat Blood Bank. In brief, to obtain a pure suspension of erythrocytes, 5 mL of whole blood was brought to 50 ml using phosphate buffered saline (PBS, pH 7.4), and centrifuged at 2500 g for 5 min at 4 °C. The supernatant was removed by gentle aspiration, and the washing was repeated until the supernatant was clear. The washed erythrocytes were finally resuspended in PBS to a final concentration of 4% (v/v). For each hemolysis assay, 400 μl of the crude extract, the chromatographic fractions or their serial dilutions in PBS were incubated with 100  μl of the erythrocyte suspension at 37 °C for 30 min in a water bath, 1 ml of PBS was added, and the assays were centrifuged at 2500 g for 5 min at 4 °C. The supernatants were transferred to 96-well microplates and the absorbance at 540 nm was determined by using a spectrophotometric microplate reader (BioTek Synergy™ HTX for the analyses of the inter- and intra-colony variation and Perkin-Elmer EnSpire for the analyses of the chromatographic separations). Positive control (100% hemolysis) and negative control (0% hemolysis) were also determined by incubating erythrocytes with DDW and PBS alone, respectively. In this study, the amount of homogenate lyses 50% of human erythrocytes in 0.5 ml after incubation at 37 °C for 30 min was defined as 1 hemolytic unit.

### Bioassay-guided column chromatography and mass spectrometry

The *Stylophora* hemolysin was partially purified using a combination of Ion Exchange Chromatography (IEX) followed by Hydrophobic Interaction Chromatography (HIC). IEX chromatography was carried out using a 18 ml Q- sepharose FF column equilibrated with 50 mM Tris- HCl, PH = 7.8, and eluted with 50 mM Tris-HCl + 1 M NaCl, PH = 7.8. The sample volume loaded on the IEX column was 3 ml and the flow rate was 12 ml/min. 1 ml fractions were collected, subjected to the hemolysis assay described above, and the active fractions were pooled as described in the results section, below. One active fraction was then adjusted to 3 M NaCl and applied to a 7.5 ml Phenyl- sepharose HP HIC column, equilibrated with 50 mM + 3 M NaCl, PH = 7.8, and eluted with 50 mM Tris-HCl, PH = 7.8. The sample volume loaded on the HIC column was 10 ml and the flow rate was 3 ml/min. The fractionation volume of the elution was 2 ml at 0%- 50% B and 5 ml at 50%-100% B. The active fractions were stored at -80◦C between each chromatography step. Proteins in the fractions after HIC were identified using Tandem MS/MS, performed at the Smoler Center for Proteomics at the Technion. Protein samples were digested by trypsin, analyzed by LC-MS/MS on a Q-Exactive tandem mass spectrometer (Thermo) and identified using the Discoverer software against all predicted proteins from the transcriptome of *Stylophora pistillata* ^76^.

### Bioinformatic analyses

Sea anemone actinoporins and Gastropod actinoporin- like sequences were retrieved from the NCBI database using BLASTp with the Stylophora hemolysin and Equinatoxin-II (EqT-II) as queries. Hydra- Actinoporin- like sequences (HALS) are those described in Glasser *et al*.^71^. Sequences of reef building organisms were retrieved similarly from the reef genome “comparative genomics” website at http://comparative.reefgenomics.org/blast/# ^76^. A second round of BLAST was performed using all of the sequences identified as discussed above against the reef genomes website, the *Nematostella vectensis* genome and the ncbi Transcriptome Shotgun Assembly (TSA) database to verify the completeness of the sequence database. Sequences with internal stop codons were removed and the sequences were aligned in using MAFFT version 7 at http://mafft.cbrc.jp/alignment/server/ ^104^ with the default parameters. The alignment was clipped at the start and end to correspond to the protein coding region of Equinatoxin-II (Fig. 5), and sequences that had low coverage (<20%) of this region, or whose alignment over this region was of extremely low quality, were removed. A full list of the actinoporins identified in the search, as well as their sequences, can be found in the Supplementary fasta file. The most appropriate model (WAG + G) was selected using ProtTest 3.2 (Darriba *et al*., 2011), a Maximum Likelihood tree was constructed in MEGA 6 with 1000 bootstrap pseudoreplicates, and a Bayesian tree was computed using MrBayes with the analysis stopped when the split frequencies were below 0.01 (~2,000,000 generations). Secondary structure prediction was performed using the PSIPRED server (http://bioinf.cs.ucl.ac.uk/psipred/).

### Statistical analyses

One-Way ANOVA was employed to compare mean values (Tukey-Kramer HDS). Data were analysed using the JMP IN® 8 statistical software (SAS Institute Inc., Cary, NC). In all analyses, statistical significance was considered at a value of P < 0.05. All means are presented as mean values ± SEM.

## Electronic supplementary material

Supplementary Table 1

Supplementary Dataset 1
