# Supplementary material for: The chemical armament of reef-building corals: inter- and intra-specific variation and the identification of an unusual actinoporin in Stylophora pistilata
Source: Sci Rep. 2018 Jan 10;8:251. doi: 10.1038/s41598-017-18355-1 (PMC5762905; doi:10.1038/s41598-017-18355-1)
Supplement: Supplementary file 1 — Supplementary Table 1 [file 41598_2017_18355_MOESM1_ESM.pdf]

The chemical armament of reef-building corals: inter- and intra-specific variation and the identification of an unusual actinoporin in *Stylophora pistilata*.

Hanit Ben-Ari[1,2,+], Moran Paz[1,+] and Daniel Sher[1*]

[1]Department of Marine Biology, Leon H. Charney School of Marine Sciences, University of Haifa, Haifa, Israel; [2]The Interuniversity Institute for Marine Sciences, Eilat, Israel.

[+] These two authors contributed equally to the study

* To whom correspondence should be addressed: Daniel Sher, [1]Department of Marine Biology, Leon H. Charney School of Marine Sciences, University of Haifa, Haifa, Israel. dsher@univ.haifa.ac.il

Supplementary information

1) Table with number of actinoporins in different cnidarian transcriptomes
2) FASTA file (separate) including the sequences of the actinoporins

Species	Number of putative actinoporins
Acropora digitifera	1
Acropora hyacinthus	0
Acropora millepora	1
Acropora palmata	1
Acropora tenuis	1
Astreopora sp	1
Favia sp	2
Fungia scutaria	1
Madracis auretenra	1
Montastraea cavernosa	3
Montastraea faveolata	2
Platygyra carnosus	4
Pocillopora damicornis	2
Porites astreoides	2
Porites australiensis	6
Porites lobata	4
Pseudodiploria strigosa	1
Seriatopora hystrix	1
Seriatopora sp	4
Stylophora pistillata	2
